# Comparative Metabolic Analysis of CHO Cell Clones Obtained through Cell Engineering, for IgG Productivity, Growth and Cell Longevity

**DOI:** 10.1371/journal.pone.0119053

**Published:** 2015-03-13

**Authors:** Camila A. Wilkens, Ziomara P. Gerdtzen

**Affiliations:** Centre for Biotechnology and Bioengineering (CeBiB), Department of Chemical Engineering, University of Chile, Beauchef 850, Santiago, Chile, 8370448

## Abstract

Cell engineering has been used to improve animal cells’ central carbon metabolism. Due to the central carbon metabolism’s inefficiency and limiting input of carbons into the TCA cycle, key reactions belonging to these pathways have been targeted to improve cultures’ performance. Previous works have shown the positive effects of overexpressing PYC2, MDH II and fructose transporter. Since each of these modifications was performed in different cell lines and culture conditions, no comparisons between these modifications can be made. In this work we aim at contrasting the effect of each of the modifications by comparing pools of transfected IgG producing CHO cells cultivated in batch cultures. Results of the culture performance of engineered clones indicate that even though all studied clones had a more efficient metabolism, not all of them showed the expected improvement on cell proliferation and/or specific productivity. CHO cells overexpressing PYC2 were able to improve their exponential growth rate but IgG synthesis was decreased, MDH II overexpression lead to a reduction in cell growth and protein production, and cells transfected with the fructose transporter gene were able to increase cell density and reach the same volumetric protein production as parental CHO cells in glucose. We propose that a redox unbalance caused by the new metabolic flux distribution could affect IgG assembly and protein secretion. In addition to reaction dynamics, thermodynamic aspects of metabolism are also discussed to further understand the effect of these modifications over central carbon metabolism.

## Introduction

Improving cell metabolism has been a common objective for researchers in the field of cell culture for many years. Previous studies have determined that cells in culture make an inefficient use of glucose, producing high levels of lactate, which has a negative effect on cell proliferation and protein synthesis [1–4]. A cell engineering approach has been proposed to improve cell metabolism, overexpressing or knocking down key genes involved in the central carbon metabolism [5,6].

A successful study to improve central carbon metabolism was carried out by Irani and collaborators. They overexpressed a copy of the yeast *pyruvate carboxylase* (PYC2) in BHK-21A cells in order to augment the pyruvate input into the TCA cycle [7]. Results of this investigation showed that after clonal selection, recombinant cells are able to achieve similar cell densities than the parental cell, while consuming less glucose and glutamine, producing less lactate, and showing a higher ATP concentration and TCA cycle fluxes. In a posterior work by the same researchers, they studied the impact of PYC2 overexpression on the production of erythropoietin by BHK-21A cells [8]. Results showed that in perfusion cultures, engineered cells were able to produce two times more recombinant protein than wild-type cells and achieved higher specific production rate. Due to the impact of these results, other investigators have studied the effect of PYC2 overexpression over other cell lines such as HEK 293, *Trichplusia ni* [9] and CHO cells [10], reaching similar positive results.

Inefficient glucose metabolism has been linked to high glucose consumption. To control this issue, media design strategies have been proposed. However, but the use of most alternative sugars does not lead to high cell density cultures [11,12]. Wlaschin and Hu proposed to overexpress the SLC2A5 gene which translates into the fructose transporter GLUT5 and use fructose as the main carbon source in CHO cells [13]. Results indicate that selected recombinant clones in fructose were able to reach higher cell densities than the parental cells in glucose. These engineered cells were characterized by a better use of the main carbon source, consuming a lower amount of carbon molecules and producing less lactate. To further investigate the impact of SLC2A5 gene overexpression, in 2010 Inoue and collaborators reported that cells derived from human myeloma overexpressing GLUT5 were able to achieve more than 1.5 times the cell density reached by wild-type cells and produce more than 2 times the amount of recombinant protein [14].

In a work by Chong and collaborators they concluded that the conversion of malate into oxaloacetate could act as a bottleneck of the TCA cycle due to malate accumulation in the extracellular media [15]. In this same work, the authors proposed to overexpress the *malate dehydrogenase II* (MDH II) gene to improve TCA cycle flux. They observed that engineered selected cells have higher ATP and NADH intracellular concentration, being able to reach almost twice the cell density that wild-type cells achieve in fed-batch cultures.

In this work we aim at gaining a better understanding of the real impact that each of these modifications has over a specific recombinant protein producing cell line. Specifically we compare cell growth, metabolic efficiency and recombinant protein production on an IgG producing Chinese hamster ovary (CHO) cell line. In order to have a clear assessment of the effect of the manipulation over culture’s performance and avoid enhancing any previous positive bias that a specific clone could have, in this work we have analyzed cell pools and not cells that have undergone best clone selection.

## Material and Methods

### Cell line and culture conditions

The IgG producing cell line CHO DP12 clone#1933 [CHO DP12, clone#1933 IL8.92 NB 28605/12] (ATCC CRL12444) was grown in adherence in T75 flasks maintained in a 1:1 mixture of Dulbecco’s modified Eagle’s medium (DMEM) and Ham’s F12 (Gibco, ME090283L1) medium supplemented with 5% FBS (Hyclone, SH30910.03), 200 nM Methotrexate (Sigma, M8407), 5 mg/L transferrin (Sigma, T8158), 1.7 μL/L 2-mercaptoethanol (Sigma, M3148-250), 0.1 ml/L ethanolamine (Sigma, E0135), 0.11 mM ascorbic acid (Sigma, A4544), 0.18 mg/L putrescine (Sigma, P5780), 29 mM sodium bicarbonate (Sigma, S4019), 28.9 mM sodium selenite (Sigma, S5261), 0.2 g/L pluronic F68 (Sigma, P1300), with a final concentration of glucose (Sigma, G5146) or fructose of 20 mM (Sigma, F3510) and glutamine 4 mM (Sigma, G1517). Selection antibiotics were added for selecting positively transfected cells and for maintaining selective pressure on transfected cultures; 680 μg/mL zeocin (Invitrogen, R250) and 250 μg/mL hygromycin (Invitrogen, 10687-010). Cells were maintained inside a CO_2_ incubator (Shel Lab, USA) at 37.0°C, with 96% relative humidity in a 5% CO_2_ enriched air atmosphere.

Growth curve experiments were performed in batch cultures with biological duplicates, monitoring their progress twice a day by determining cell density and sampling the supernatant for further analysis. Cells were inoculated at a concentration of 5.5×10^4^ [cells cm^-2^] (equivalent to 0.14×10^6^ [cells mL^−1^]) with cells from the mid exponential phase of growth. Cell number and viability were determined by the trypan blue exclusion method. After cell counting the supernatant was spun briefly and frozen at -20°C before metabolites analysis.

### Metabolite determination

Glucose, fructose and lactate concentrations were determined with the appropriate kit as recommended by the supplier (Randox, GL364; Biovision, K619; Randox, LC2389) and glutamine was determined with a YSI analyzer. IgG concentration was quantified by ELISA. Briefly, the ELISA protocol used goat anti-human IgG Fc-specific (Sigma-Aldrich, Sigma-Aldrich, I3391) and mouse anti-goat IgG Alkaline phosphatase (Sigma-Aldrich, A2064) as primary and secondary detection antibodies. P-nitro-phenyl phosphate was used as the enzyme’s substrate (Sigma-Aldrich, N1891). The calibration curve was constructed using human IgG (Pierce, PI31879) as standard. IgG concentration was determined by absorbance reading at 405 nm. NAD^+^/NADH ratios were measured using Biovision’s NAD^+^/NADH Quantification Colorimetric Kit (Biovision, K337-100) following the protocol provided by the supplier.

### Vectors, transfection and selection

Vector construction for overexpressing the fructose transporter was carried out as described in the work by Wlaschin and Hu using pcDNA3.1(+) with zeocin resistance (Invitrogen, V860-20) [13]. For overexpressing the *pyruvate carboxylase* gene the pCMVSHE-PYC2 vector used by Irani and collaborators [7] was cotransfected in a 10:1 ratio with a plasmid containing PGK-hygromycin cassette for selection, provided by GEMF at M.D. Anderson Cancer Center. Overexpression of the *malate dehydrogenase II* gene was achieved using the vector constructed by Chong and collaborators [15] where the MDH II gene was cloned into pcDNA3.1(-) with hygromycin resistance for selection. The expression of all these genes was controlled by the CMV constitutive promoter. All constructs in this work were verified by PCR and sequencing.

To determine that the changes seen in the studied clones are due to gene overexpression and not to the plasmids or the expression of different resistance genes, control clones were also constructed by transfecting the parental cell line with the empty vectors pcDNA3.1(+) zeocin, pcDNA3.1(-)hygro and PGK-hygromycin cassette.

Cells were transfected employing polyethylenimine (PEI) (Polysciences, Inc., 24765-2) using a modified version of the protocol described by Jones and collaborators [16]. The day prior to transfection 0.4×10^6^ [cells mL^−1^] were inoculated in a 60 mm petri dish. The transfection complex was prepared by mixing 5 μg of DNA with 14 μL of a 1[μg/μL] PEI solution, adding serum free media to complete a volume of 625 μL. The complex was incubated for 20 min at room temperature and then added to the cells. Final volume was completed with 1.875 mL of serum free media. At 4 h post transfection all media was replaced with fresh media supplemented with 5% FBS. Selection of stable clones began after 72 h after transfection by reducing FBS to 2% and adding the appropriate antibiotic concentration, previously determined by a cell death curve. After 1 week cells that had the desired vector were positively selected and FBS was increased to 5%. Cells were maintained under selective pressure for the duration of experiments.

### Specific rate determination

The specific rate of production or consumption of cell metabolites was calculated from a cumulative curve constructed by adding the differential amount of a metabolite produced or consumed between two consecutive time points in the culture. The specific rates were calculated as the derivative of the curve fitting this data divided by the cell density evaluated in each time point. This method ensures small error propagation from measurement errors. The value of *ΔL/ΔHexose* is defined as -*q*_*Lactate*_/*q*_*Hexose*_ and it was calculated as the quotient between the two specific rates.

## Results and Discussion

Different pools of engineered clones were obtained through transfection. CHO cells overexpressing PYC2, MDH II and fructose transporter are referred as CHO PYC, CHO MDH and CHO FrcTr respectively. The study of these cell pools was done by comparing clones’ performance in culture against parental cells grown in glucose- and fructose-based media. In this work we aimed at studying CHO PYC and CHO MDH with glucose as their main carbon source and compare them only to CHO cells grown in glucose based media. CHO FrcTr cells are grown in fructose-based media and are compared to both control cultures.

To prove that the results from engineered cells can be attributed to gene overexpression and not to the metabolic burden caused by transfected plasmids and/or the resistance gene expression, control clones expressing only the antibiotic resistance gene were constructed as explained in the Material and Methods section. These cell clones are referred as CHO PGK-Hygro and CHO pcHygro CHO pcZeo and their growth, metabolism and IgG productivity in glucose based media are compared to those of the parental cell line in the same culture condition.

The parental cell line used in this work is a low IgG producer clone. This characteristic was selected in order to assure that protein synthesis would not face limiting factors such as amino acid depletion or endoplasmic reticulum stress due to unfolded protein response. This enables us to isolate the effect of each modification on protein productivity. In order to study the overall effect of each modification over a population of CHO cells, no single cell selection was performed for any of the constructed cells.

### Engineered clones cultures’ performance

**Fig 1:**
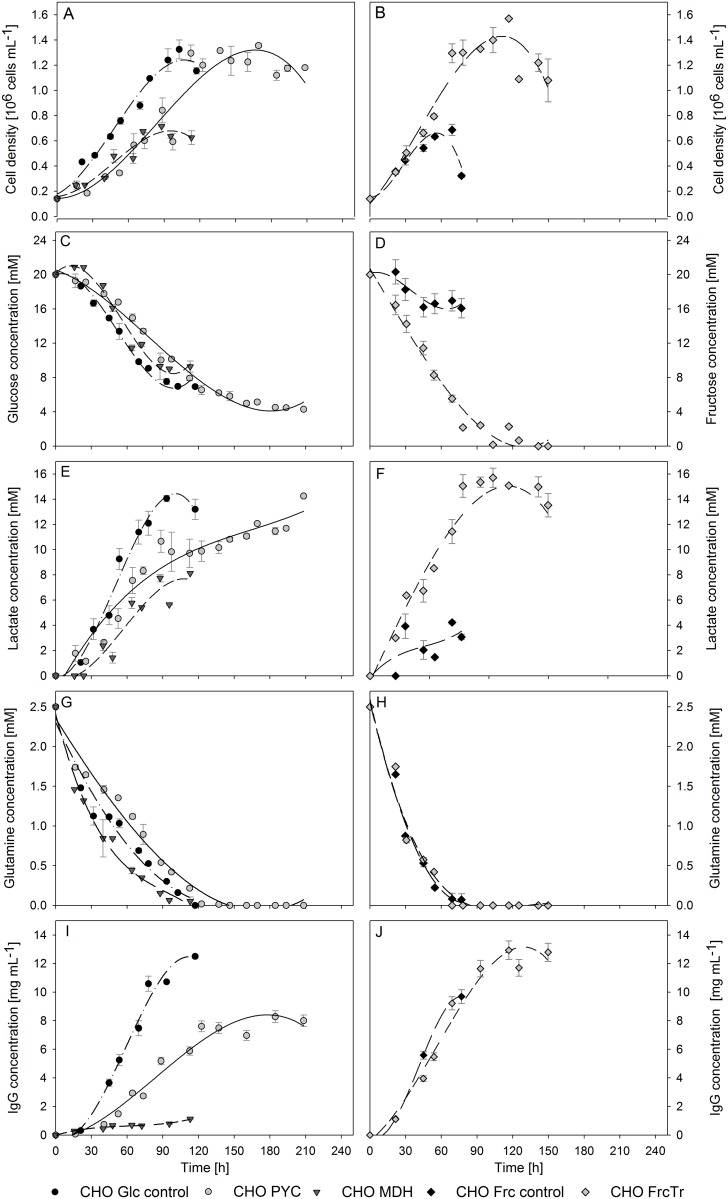
Batch cultures of different CHO cell engineered clones as a function of time. Graphs to the left describe cultures that use glucose as the main carbon source and graphs to right depicts cultures that use fructose as their main carbon source. (●) CHO Glc control; (●) CHO PYC; (▼) CHO MDH; (♦) CHO Frc control; (♦) CHO FrcTr. A, B: Viable cells; C, D: main carbon source concentration; E, F: lactate concentration; G, H: glutamine concentration; I, J: IgG concentration.

Fig. 1 shows cell growth, glucose or fructose consumption, lactate production, glutamine uptake and IgG production for CHO PYC, CHO MDH and CHO FrcTr clones compared to parental cell line cultures. Table 1 presents the parameters that describe the cultures’ growth, metabolism and productivity associated to the data presented in Fig. 1. Fig. 1A and B and Table 1 provide information regarding growth of the studied clones. Wild-type CHO cells in glucose-based media are able to sustain high cell viability for 120 h, proliferating at a maximum rate (μ_max_) of 1.65×10^-2^ h^-1^ for 62 h, and reaching a cell density of 1.2×10^6^ [cells mL^-1^]. As expected, when wild-type CHO cells are grown using fructose as their main carbon source their growth is clearly altered, as cultured cells have a lifespan of only 65 h, while exhibiting a μ_max_ similar to the one observed for control cells grown in glucose. The exponential phase lasted for only 23 h leading to a low cell density of 0.65×10^6^ [cells mL^-1^]. Culture performance of engineered cells differs from the one exhibited by parental cells. CHO PYC cells have an extended lag phase, followed by an 81 h long exponential growth phase characterized by a μ_max_ of 1.76×10^-2^ h^-1^. By the end of this period CHO PYC cells have reached the same cell density as the control experiment in glucose. After this, the culture is capable of sustaining a long stationary phase before death. CHO cells overexpressing the MDH II enzyme exhibit a higher μ_max_ than control cells but with a shorter exponential phase, which explains the low cell density achieved. Finally, CHO FrcTr cells were grown with fructose as the main carbon source and overexpression of the fructose transporter translated into the highest μ_max_ of the studied cells. This is the only culture that can be considered as statistically different from the glucose control, with a p-value of 0.023. CHO FrcTr cells reached the highest cell density (1.5×10^6^ [cells mL^-1^]) achieved by any of the studied clones.

**Table 1:** Parameters for cell growth, ΔL/ΔH^a^, IgG’s specific productivity and their percentage variation vs. their respective control cultures.

	Parental CHO cells Glc control	Parental CHO cells Frc control	CHO PYC	CHO FrcTr	CHO MDH
**Exponential phase length [h]**	62	23	70	62	50
			12.9%	0%	-19.4%
**μ_max_** **[10** ^**-2**^ **h** ^**-1**^ **]**	1.65±0.07	1.62±0.03	1.76±0.25	2.35±0.16	1.72±0.25
			6.7%	42.4%	4.2%
**ΔL/ΔH**	1.042±0.15	0.629±0.14	0.79±0.12	0.653±0.07	0.559±0.08
			-24.2%	-37.3%	-46.4%
**Specific IgG production [mg IgG h** ^**-1**^ **10** ^**–6**^ **cells**	2.30×10^-4^	3.79×10^-4^	1.08×10^-4^	1.99×10^-4^	1.71×10^-5^
			-53.0%	-13.5%	-92.6%

^a^H stands for hexose, depending on the culture’s conditions it can be either glucose (G) or fructose (F).

Results regarding glucose, fructose and glutamine uptake, and lactate production are shown in Figs. 1C, D, E, F, G and H and in Table 1. By the end of the culture’s lifespan, parental cells grown in glucose consumed nearly 65% of the available glucose and all the available glutamine, and produced a total of 14 mM of lactate with a ΔLactate/ΔGlucose (ΔL/ΔG) of 1.04. Parental cells grown in fructose-based media consumed only 20% of the available fructose and all the glutamine, while producing only 4 mM on lactate with a ΔLactate/ΔFructose (ΔL/ΔF) of 0.63.

Results show that engineered cells have a different metabolism than the one exhibited by control cells. CHO PYC cells are able to consume glucose at a high rate during the first 100 h of culture producing lactate with a ΔL/ΔG of 0.79, which indicates a more efficient metabolism with lower lactate production per glucose consumed. At this time glutamine is depleted. In the following stage of the culture, CHO PYC cells consume glucose at a lower rate, which is apparent from the change in the slope of the curve in Fig. 1C. During this low glucose consumption period cells neither consume nor produce any lactate, and are able to sustain high viability without using glutamine as a main carbon source. CHO MDH cells consume 60% of the available glucose, all available glutamine and produce 8 mM of lactate with a ΔL/ΔG of 0.56, showing the highest metabolic efficiency among the studied clones; this ΔL/ΔG value has a significant statistical difference with the glucose control experiment with a p-value of 4.7×10^-5^. Lastly, overexpression of the fructose transporter in the CHO FrcTr cells enabled them to consume nearly 90% of the fructose present in the culture media at a high rate, as can be observed from to the steep slope of the curve in Fig. 1D. This higher fructose consumption led to a high lactate synthesis producing 16 mM of lactate, but the ΔL/ΔF achieved is only of 0.65, which indicates a more efficient metabolism than control cells in glucose (p-value 0.018). Glutamine depletion in this culture occurred after nearly 70 h of culture, but cells were able to continue proliferating after this for 80 more hours.

Differences regarding IgG production can be observed in Table 1 and Fig. 1I and J. Control cells grown in glucose and fructose were able to produce 12.5 and 9.7 mg/L of IgG respectively at a specific rate of 2.3×10^-4^ and 3.79×10^-4^ [mg h^-1^ 10^-6^ cells]. Unlike what has been reported already regarding cells overexpressing the PYC2 gene, the engineered CHO cells obtained were unable to exceed control cells productivity, producing 7 mg/L at a specific rate of 1.08×10^-4^ [mg h^-1^ 10^-6^ cells] during the exponential growth phase, with no significant recombinant protein synthesis observed during the stationary phase. To this date the effect of MDH II overexpression on productivity has not been reported. Our results evidence a drastic decline in productivity, as CHO MDH cultures reached a final production of 1 mg/L at a specific rate of 1.71×10^-5^ [mg h^-1^ 10^-6^cells]. CHO FrcTr cells were able to produce a similar amount of IgG at the end of culture’s lifespan in comparison to control culture in glucose, reaching an IgG concentration of 12.8 mg/L, but its specific production rate was decreased reaching only 1.99×10^-4^ [mg h^-1^ 10^-6^cells]. This means that increased volumetric production of CHO cells grown in fructose based media observed would be caused mainly by the increase in cell density and culture’s lifespan and not due to an improvement in the cells’ specific production rate.

Better culture strategies can be proposed based on our understanding of the cultures’ performance. The results presented here show that CHO PYC and CHO MDH cultures exhibit no significant improvement in terms of exponential growth rate, culture’s lifespan or specific productivity; therefore they are not better candidates for industrial production than the parental cell line. On the other hand, CHO FrcTr batch cultures showed that there was no significant statistical difference with the control CHO cells grown in fructose based media, but there were significant differences in comparison to CHO cells grown with glucose as the main carbon source. CHO FrcTr cells were able to match glucose control culture’s performance in terms of volumetric IgG production and surpass parental cells’ production in fructose-based media. IgG production by CHO FrcTr clones in fructose was improved mainly due to biomass increase, therefore the use of fed-batch or perfusion reactors to further improve volumetric production could be limited by the maximum cell density that these cells can achieve in these reactors.

### Analysis of the potential metabolic burden contribution to the observed clones’ behavior

Control cultures were performed in order to determine that all the differences stated above were due to the overexpression of the gene of interest and not from the metabolic burden caused by either the presence and replication of the vector, or the expression of the resistance gene or addition of antibiotic. For this analysis, the percentage variation of important culture parameters for engineered clones and control cell cultures observed are summarized in Table 1 and Table 2. Exponential phase length was not impacted by transfection of empty vectors whereas engineered cells’ growth span changed between -19.4 and 12.9%. The differences in μ_max_ between control cultures and the parental cell culture range between 4.2 and 8.3%, while engineered cells present differences between 4.2 and 42.4%. Glucose consumption and lactate production were also not significantly altered in control cell cultures, exhibiting variations of ΔL/ΔG between -11.6 and -2.3%, while engineered clones’ ΔL/ΔG ranges from -46.6 to -24.2%. Lastly, specific productivity for cells with empty vectors is similar to parental cells’ exhibiting changes from -11.4 to 18.7%, which is not as significant as the changes of specific production of engineered clones that goes from -13.5 to 92.6%. All these differences indicate that the changes in parameters observed experimentally are most likely caused by the overexpression of the studied genes.

**Table 2:** Percentage variation for control cultures’ parameters compared to a parental cell culture

	CHO pcZeo	CHO PGK-Hygro	CHO pcHygro
**Exponential phase length**	0	0	0
**μ**_**max**_	8.3	4.2	8.3
**ΔL/ΔH**	-2.3	-11.6	-9.3
**Specific IgG production**	18.7	17.4	-11.4

### Effects on central carbon metabolism

**Fig 2:**
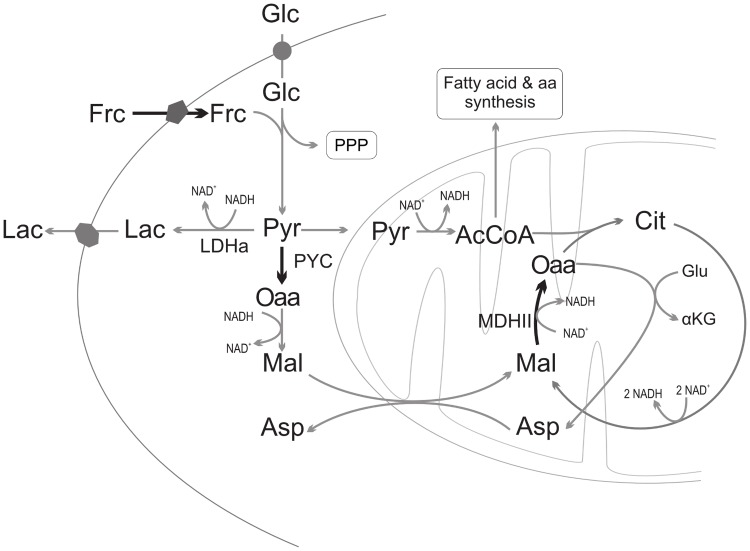
Impact of each of the modifications over CHO cells’ metabolism. Reactions catalyzed by overexpressed enzymes are represented by bold lines. Glc: Glucose, Frc: Fructose, Cit: Citrate, AcCoA: Acetyl CoA, Mal: Malate, Oaa: Oxaloacetate, Asp: Aspartate, Pyr: Pyruvate, Lac: Lactate, αKG: α ketoglutarate, LDHa: Lactate Dehydrogenase a, PPP: Pentose Phosphate Pathway.

The effect of PYC2 overexpression has a clear impact over the cultures performance. In previous works done on BHK cells [7,8], cultures reached higher cell densities, had an extended lifespan and cells exhibited a more efficient metabolism producing less lactate per glucose consumed and were able to maintain high viability without glutamine consumption. In another publication by Fogolin and collaborators, PYC2 was overexpressed in CHO cells and they reported a drop in cell density, but cells also exhibited improvements in lifespan, metabolic efficiency and recombinant protein synthesis [10]. Results obtained in this work regarding CHO PYC cells show a reduction of the total recombinant protein production and the specific rate at which it is produced. Fig. 2 illustrates some of the most important metabolic pathways that are involved in the central carbon metabolism in animal cells.

PYC enzyme converts pyruvate into oxaloacetate, which then turns into malate. This molecule is afterwards transported into the mitochondrial matrix through the malate-aspartate shunt. From the diagram in Fig. 2 it is possible to observe that the first consequence that can be inferred from overexpressing PYC is that cells would have a higher input of reducing power from the cytosol into the mitochondria due to the higher flux through the Malate-Aspartate shuttle, therefore increasing the cell’s energy metabolism, which could explain cultures extended lifespan. Table 3 shows a comparison of NAD^+^/NADH ratios of cultured cells. It is possible to observe that CHO PYC’s NAD^+^/NADH ratio compared to the control culture in glucose based media is significantly lower (p-value 0.003), evidencing an enhanced energy metabolism. The second impact is a reduction in the available pyruvate for other metabolic pathways. As a previous work by the authors showed, lactate synthesis is related to pyruvate accumulation inside the cell [17], PYC2 overexpression should lead to a reduction of pyruvate concentration in the cytosol therefore reducing lactate synthesis and improving the cells ΔL/ΔG ratio. Finally, CHO PYC cells exhibit a higher growth rate than control cells, which has been shown to negatively influence protein synthesis.

**Table 3:** NAD+/NADH ratios of studied cultures

Cell Cultures	NAD^+^/NADH
Parental CHO cells Glc control	17.12±2.04
Parental CHO cells Frc control	4.19±1.05
CHO PYC	3.66±0.95
CHO FrcTr	3.64±0.71
CHO MDH	8.57±1.84

Previous works indicate that cells growing at lower rates are able to produce recombinant proteins at higher rates than fast growing cultures due to increased resource availability [18,19]. In this work we propose a second mechanism through which faster proliferation can affect protein synthesis. Cells are dependent on NADPH for biosynthetic pathways and for the generation of reduced glutathione. By increasing NADPH use for proliferation, less of this nucleotide would be available for maintaining appropriate GS/GSSG ratios in the cell, specifically in the endoplasmic reticulum. Borth and collaborators showed that intracellular assembly of light and heavy chain is a major limiting factor for overall cell specific IgG productivity [20]. We believe that for the cells analysed in this study, when cellular NADPH requirements increase towards anabolic pathways a GS/GSSG unbalance occurs in the endoplasmic reticulum that affects IgG assembly and therefore reduces protein production. In Irani’s and Fogolin’s works the recombinant protein produced were erythropoietin and rhGM-CSF respectively, which consist in only one polypeptide chain [21,22] and therefore in those cases protein synthesis may not have encountered assembly problems.

Cells overexpressing the MDH II enzyme are able to alleviate a bottleneck of the TCA cycle that is the conversion of malate into oxaloacetate. The work by Chong and collaborators showed that by augmenting MDH II activity, the flux through the Malate-Oxaloacetate (Mal-Oaa) step of the cycle increases [15]. As a direct result of this, Chong reported an increase of the NADH production that improves the cells energy metabolism; also supported by data in Table 3, where a lower NAD^+^/NADH ratio can be seen (p-value 0.03). A second effect is that by rising oxaloacetate concentration the condensation reaction between oxaloacetate and acetyl CoA (AcCoA) would exhibit higher rates and therefore all fluxes through the TCA cycle should be improved. By increasing mitochondria’s acetyl CoA’s requirements there is less of this key metabolite for other metabolic pathways such as fatty acid synthesis and other molecules, which could have a direct impact over biomass production, therefore, explaining the lower cell proliferation observed in CHO MDH cultures. Cellular requirements for acetyl CoA could also explain the lower lactate production observed as more pyruvate is converted into acetyl CoA instead of lactate. Finally, the reduced IgG production could be explained by a GS/GSSG unbalance, but in this case it is expected to be due to lower fluxes in the pentose phosphate pathway, which as Boada and collaborators showed, can be directly correlated to NADPH reduction [23].

As shown by the results in Table 1, CHO cells overexpressing the GLUT5 transporter are able to consume fructose at higher rates than the glucose uptake of parental cells, and continue cell proliferation without glutamine in the culture media. This would enable a higher pyruvate production rate from the glycolytic pathway and therefore lead to pyruvate accumulation, as suggested by previously published results [17]. As indicated above, when pyruvate concentration inside the cell increases it is expected to observe a higher lactate synthesis rate; nevertheless carbon metabolism of CHO FrcTr grown in fructose proved to be more efficient, with lower lactate synthesis than that of CHO cells in glucose, as shown by the lower ΔL/ΔF ratio observed for CHO FrcTr. Table 3 shows that CHO FrcTr cells exhibit a lower NAD^+^/NADH ratio compared to control CHO cells grown in glucose (p-value 0.014) and similar to control cells in fructose based media. Decreased NAD^+^/NADH levels are consistent with pyruvate accumulation and lower lactate synthesis since glucose or fructose degradation via glycolytic pathway produces NADH and the Pyruvate-Lactate (Pyr-Lac) reaction oxidizes NADH; therefore higher glycolytic fluxes and lower Pyr-Lac fluxes would lead to increased NADH levels. CHO FrcTr cultures had an improved total IgG production compared to parental cells, but this fact can only be attributed to the higher cell density achieved by cultures, since specific IgG production rate was reduced compared to the control. This drop can be explained by higher NADPH requirements altering the GS/GSSG balance in the endoplasmic reticulum and negatively affecting IgG assembly in a similar way as proposed above for CHO PYC cells.

### Thermodynamic implications

Modifications over any enzyme’s expression will likely lead to changes in the intracellular pools of the metabolites involved in the reactions catalyzed by these enzymes [10,24,25], therefore changing the reaction’s available Gibbs free-energy. Table 4 shows the standard free-energies changes (ΔG’^0^) for some important reactions discussed in this work and an approximation to their equilibrium constants (K’_eq_) [26]. When the PYC enzyme is overexpressed it is expected for the cytoplasmic and mitochondrial concentration of pyruvate to decrease as a result of this modification.

**Table 4:** Standard Gibbs free-energies for studied reactions and their equilibrium constants

Reaction	ΔG’^0^ [kJ mol^-1^]	K’_eq_
Pyr + CoA-SH + NAD^+^ → AcCoA + NADH + CO_2_	-33.4	7×10^5^ >
Oaa + AcCoA + H_2_O → Cit + CoA-SH	-32.2	4×10^5^ >
Mal + NAD^+^ ↔ Oaa + NADH	29.7	6×10^–6^ >
Pyr + NADH ↔ Lac + NAD^+^	-25.1	2×10^4^ >

^>^ Lower limit value

From the data shown in Table 4 it is possible to observe that the Pyruvate-Acetyl CoA (Pyr-AcCoA) reaction has a highly negative Gibbs standard free-energy, indicating that the reaction is thermodynamically favorable towards acetyl CoA synthesis with a high K’_eq_. The Pyr-AcCoA reaction’s Gibbs free-energy is given by the equation:

$$\Delta G=\Delta G{'}^{0}+RT\cdot \text{ln}\left(\frac{\left[AcCoA\right]\left[NADH\right]\left[CO_{2}\right]}{\left[Pyr\right]\left[NAD^{+}\right]\left[CoA-SH\right]}\right)$$ (1)

Based on information available in literature it is possible to estimate the free-energy for this reaction for mammalian cells in culture. The mitochondrial NADH/NAD^+^ ratio has been reported as 0.77:1, the Coenzyme A/Acetyl CoA (CoA-SH/AcCoA) is 0.627:1 [27], pyruvate concentration is 0.085 mM [28,29] and CO_2_ concentration is 11.25 mM [30,31]. The resulting average Gibbs free-energy for this reaction in mammalian cells is -20.3 kJ mol^-1^. If the available pyruvate for the reaction were reduced by tenfold, the available free-energy would be reduced by 5.9 KJ mol^-1^, making it less favorable. Even in this scenario the Pyr-AcCoA reaction is spontaneous and thermodynamically favorable, so no inconsistencies are found in this respect.

Regarding the thermodynamics of lactate synthesis in the context of PYC2 overexpression a similar analysis can be made. According to published data the NAD^+^/NADH ratio in the cytoplasm is 700:1 [32] and the Lactate/Pyruvate ratio is 19.5:1 [33], which leads to an estimated Gibbs free-energy for the reaction of -0.56 KJ mol^-1^. Wild-type CHO cells in culture show a reversed LDH reaction evidenced by lactate’s consumption. In a previous study by the authors, lactate synthesis and uptake are proposed to be determined by pyruvate’s concentration [17]. Due to this reported evidence, a low Gibbs free-energy would be expected in order for this reaction to reverse its flux under low pyruvate conditions. As seen from CHO PYC culture’s performance, lactate was synthesized throughout the exponential growth phase. This indicates that initially the glucose consumption rate is high enough to produce pyruvate at a high enough rate to feed the different metabolic pathways. A decline in pyruvate concentration leads to a reduction in the available free-energy for the reaction, leading to a lower lactate production rate.

As shown in Table 4 the standard free-energy for the Mal-Oaa reaction is positive, making this a limiting reaction in the TCA cycle, which is consistent with previously reported results [15]. In order for the reaction to proceed in the direction of oxaloacetate synthesis, the concentration of malate has to be several orders of magnitude higher than oxaloacetate as shown in both experimental and computer modeling studies [34,35]. Overexpression of MDH II is expected to increase the reactions rate, therefore increasing oxaloacetate’s accumulation in the mitochondria, which should augment the condensation rate for the reaction catalyzed by citrate synthase. Considering an average citrate and oxaloacetate mitochondrial concentration of 3.1 and 0.003 mM respectively [28,36], the average Gibbs free-energy for this reaction is -13.1 kJ mol^-1^. If the concentration of oxaloacetate would increase 10 times, the available free-energy would be -19.0 kJ mol^-1^ boosting the synthesis of citrate and therefore improving the TCA cycle from a thermodynamic point of view.

Results shown in the previous section indicate that cells that overexpress a fructose transporter are able to consume fructose at a much higher rate than wild-type cells in fructose based media. As a direct result of this, cells produce pyruvate at a higher rate, leading to pyruvate accumulation. In this scenario, with higher pyruvate concentrations, the Gibbs free-energy available for the reactions that yield lactate and acetyl CoA would be reduced by 5.9 kJ mol^-1^ each. This decrease in the available free-energy enhances the flux of carbon towards lactate and acetyl CoA production. This is consistent with CHO FrcTr cultures’ performance, where lactate synthesis is enhanced in comparison to wild-type cells. The enhanced lifespan displayed by the cells could be associated with an improved energy metabolism.

## Conclusions

All modifications studied in this work were aimed at enhancing the cultures performance by improving the cells central carbon metabolism as a result of augmenting the flux through bottleneck reactions or reducing the flux through a reaction that yields an unwanted metabolite. In previous works where these modifications were proposed, experiments were carried out in different cell lines, expressing different proteins and grown in different culture conditions, therefore results could not be compared directly. In this work we present the first comparison of the application of different metabolic engineering strategies to mammalian cells.

Each of the studied modifications had a different effect over the cultures performance. Overexpression of PYC2 enabled cells to achieve a higher maximum growth rate and a more efficient metabolism, but product synthesis was reduced. When MDH II was overexpressed exponential growth rate was also improved in comparison to the control culture, but due to a short exponential phase cells were not able to reach high densities. Even though CHO MDH cells presents the lowest production of lactate per glucose consumed they were neither able to produce IgG at an increased rate, nor to improve the volumetric production of recombinant protein. When cells overexpressed the fructose transporter and were cultured in fructose-based media, they improved their growth in the exponential phase, reaching higher cell densities and increasing their recombinant protein volumetric production. Based in previously published results it was expected to find high improvement in all cultures studied in this work, mostly due to the improvement in their use of the main carbon source [37]. We propose that cells overexpressing PYC2, MDH II or GLUT5 might not present problems in the polymerization of the peptidic chains, but the overexpression of these proteins may cause an unbalance in the NADP/NADPH and GS/GSSG, which would explain the observed experimental results. As shown in S1 Fig., an NADP^+^/NADPH unbalance leads to an unfavorable redox state for light and heavy chain assembly, which would in turn cause a reduced protein production due to lower protein secretion.

We extended the analysis of the effect of the metabolic modifications performed in this work, to the examination of the implications of these changes in the thermodynamics of the metabolic network. Our analysis showed that by changing the expression of each of the studied enzymes, key reactions in the metabolism could be thermodynamically favored or limited. When PYC2 is overexpressed pyruvate accumulation lowers, therefore decreasing the amount of free-energy available for the lactate synthesis reaction. The resulting malate increment in the mitochondria makes the Mal-Oaa reaction thermodynamically more favorable increasing the reaction’s rate, NADH synthesis, and improving cells’ energy metabolism. On the other hand, MDH II overexpression enhances oxaloacetate production. This increase in turn increments the available Gibbs free-energy for the condensation reaction through which carbon molecules enter the TCA cycle. CHO FrcTr cells are able to produce more pyruvate. Higher pyruvate levels lead to an increase in available free-energy for lactate and acetyl CoA synthesis.

In conclusion, in this work we were able to successfully compare the effect of different modifications and evaluate their suitability for industrial use. Each of these modifications has an important impact over carbon metabolism improving the ΔL/ΔHexose that each culture exhibits and reducing NAD^+^/NADH ratios. The discussions from a metabolic and thermodynamic point of view presented in this work support previous conclusions regarding improvement of the energy metabolism of these cell modifications. In addition, we explain the drop in productivity that three of the studied clones exhibit by linking the new metabolic flux distribution to changes in the redox state, which have a detrimental effect on IgG’s assembly, therefore reducing its secretion. The impact of these modifications on protein synthesis might be different for cells producing single chain recombinant proteins. Due to the complexity of cellular metabolism, the overall effect of a given modification on the central carbon pathway is difficult to predict. However, our results suggest that by improving energy metabolism and the flux into the TCA cycle, it is possible to improve the cultures performance, provided that appropriate redox conditions can be maintained.

## Supporting Information

S1 Fig Effect of the different modifications over redox state and its effect over protein assembly. Green and red text and arrows indicate an increase or decrease in pathway flux or molecule synthesis. NAD^+^_CIT_: Cytoplasmic NAD^+^; NADH_MIT_: Mitochondrial NADH; E.R.: Endoplasmic reticulum.
(TIF)
