# Supplementary figures and images for: Comparative Metabolic Analysis of CHO Cell Clones Obtained through Cell Engineering, for IgG Productivity, Growth and Cell Longevity
Source: PLoS One. 2015 Mar 13;10(3):e0119053. doi: 10.1371/journal.pone.0119053 (PMC4358941; doi:10.1371/journal.pone.0119053)

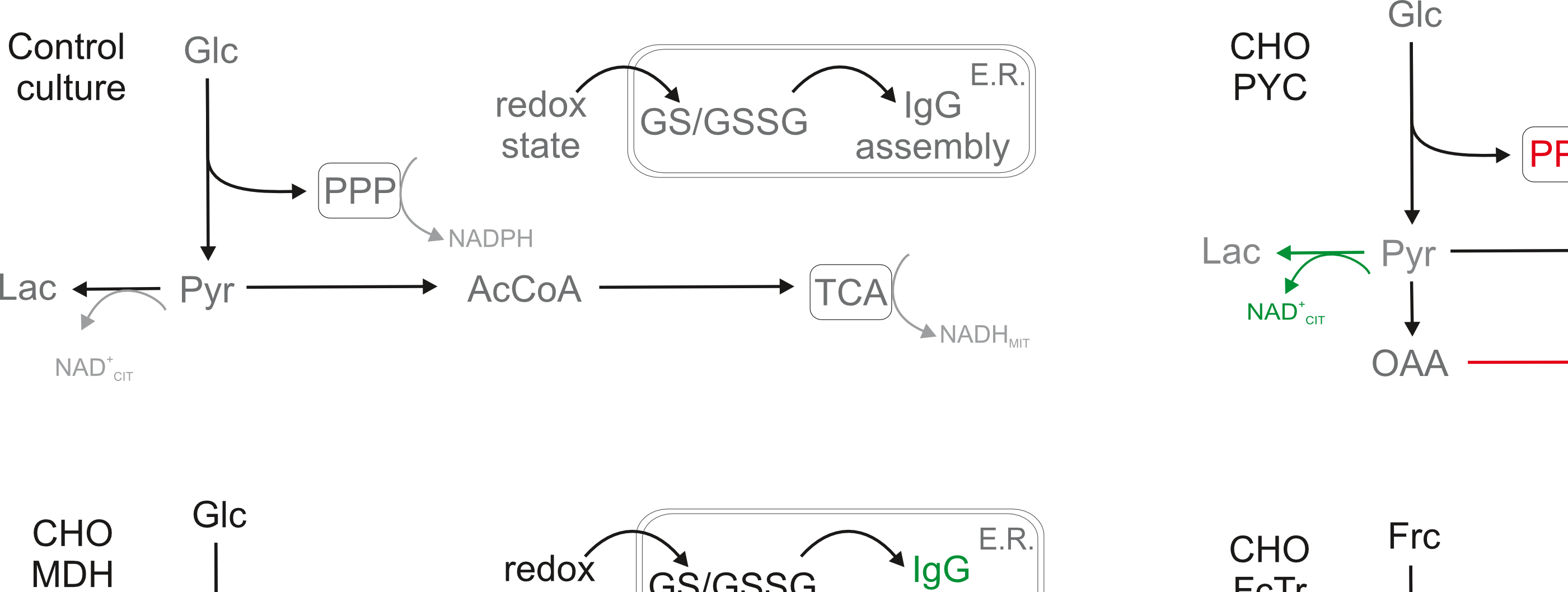

Supplement: S1 Fig — Green and red text and arrows indicate an increase or decrease in pathway flux or molecule synthesis. NAD+ CIT: Cytoplasmic NAD+; NADHMIT: Mitochondrial NADH; E.R.: Endoplasmic reticulum. (TIF) [file pone.0119053.s001.tif]
